# Validation Study of the Iranian Version of Minnesota Living With Heart Failure Questionnaire (MLHF‐Q): A Cross‐Sectional Study

**DOI:** 10.1002/hsr2.70396

**Published:** 2025-02-03

**Authors:** Majideh Heravi‐Karimooi, Razieh Bandari, Somayeh Eskandari, Sahar Semnani, Nahid Rejeh, Ali Montazeri

**Affiliations:** ^1^ Elderly Care Research Center, Faculty of Nursing & Midwifery Shahed University Tehran Iran; ^2^ Social Determinants of Health Research Center Semnan University of Medical Sciences Semnan Iran; ^3^ Arash hospital Tehran University of Medical Sciences Tehran Iran; ^4^ Cardiovascular Research Center Rajaie Cardiovascular Institute Tehran Iran; ^5^ Population Health Research Group, Health Metrics Research Centre, Iranian Institute for Health Sciences Research, ACECR Tehran Iran; ^6^ Faculty of Humanity Sciences University of Science &Culture Tehran Iran

**Keywords:** heart failure, Minnesota living with heart failure questionnaire, psychometric, quality of life, reliability, validity

## Abstract

**Background and Aims:**

The Minnesota Life with Heart Failure Questionnaire (MLHF‐Q), a globally recognized instrument, is widely used to assess the quality of life in heart failure (HF) patients. This study, conducted in Iran, investigated the psychometric properties of the Persian version of the MLHF‐Q and contributed to the global understanding of the quality of life assessment of HF patients.

**Methods:**

In this methodological study, the MLHF‐Q was meticulously translated from English to Persian using a rigorous forward‐backward translation process. Subsequently, the Persian version was comprehensively assessed for content and face validity, structural validity (through exploratory and confirmatory factor analysis), reliability, and stability.

**Results:**

Five hundred twenty‐seven heart failure patients participated in the study, with a mean age of 57.9 years (SD = 13.54). Exploratory factor analysis validated a three‐factor structure for the scale, and confirmatory factor analysis showed satisfactory model fit indices (RMSEA = 0.08, CFI = 0.92, TLI = 0.91, IFI = 0.93). The questionnaire had exceptional internal consistency and reliability, with a Cronbach's alpha coefficient of 0.97 and an intraclass correlation coefficient (ICC) of 0.98, instilling confidence in our results' robustness.

**Conclusion:**

The questionnaire used to measure the quality of life of heart patients is very accurate and reliable. It can evaluate the quality of life of these patients in three main sections (researchers, doctors, and patients).

**Abbreviations**
AF: Atrial Fibrillation
AVE: Average Variance Extracted
CABG: Coronary Artery Bypass Graft Surgery
CFA: Confirmatory Factor Analysis
CFI: Comparative Fit Index
CHFQ: Chronic Heart Failure Questionnaire
CMIN/DF: Minimum Discrepancy Function by degrees of freedom divided
CR: Construct Reliability
DASS‐21: Depression, Anxiety, and Stress Scales‐21
EFA: Exploratory Factor Analysis
GFI: Goodness of Fit Index
ICC: Intraclass Correlation Coefficients
IFI: Incremental Fit Index
KMO: Kaiser–Meyer–Olkin
KCCQ: Kansas City Cardiomyopathy Questionnaire
LBBB: Left Bundle Branch Block
LVD‐36: Left Ventricular Dysfunction questionnaire
LVEF: Left Ventricular Ejection Fraction
MLHF‐Q: Minnesota Living with Heart Failure Questionnaire
MSV: Maximum Variance
NYHA: New York Heart Association
PCI: Percutaneous Coronary Intervention
PCFI: Parsimonious Comparative Fit Index,
PNFI: Parsimonious Normed Fit Index
RBBB: Right Bundle Branch Block
RMSEA: Root Mean Square Error of Approximation
SEM: Structural Equation Modeling
SIP: Sickness Impact Profile
SF‐36: Short Form Health Survey
TLI: Tucker –Lewis Index
VT: Ventricular Tachycardia

## 1 Introduction

Heart failure, a severe heart disease, is a significant cause of mortality and morbidity in many countries, especially in low‐ and middle‐income countries [1]. The prevalence of heart failure is not uniform and ranges from 0.4% to 4.3% in the general population and from 2% to 20% in people over 75 years old [2]. In Iran, the prevalence of heart failure is 8%, and the 1‐year mortality rate is approximately 32% [3].

Risk factors for heart failure include several demographic, biological, and disease factors, including heart attack, coronary artery disease (CAD), diabetes, hypertension, obesity, and valvular heart disease. However, it is essential to note that the mortality rate of heart failure patients is significant, and approximately 50% of patients do not survive more than 5 years after diagnosis. This means that almost half of people who develop heart failure will die within 5 years [4].

Unfortunately, self‐care practices among heart failure patients in Iran are insufficient and need to be improved. A previous study showed that people with heart failure have a lower quality of life than the general population, which is due to severe clinical symptoms, impairment, and hospitalization. These patients usually reduce their daily life activities due to shortness of breath, lethargy, fatigue, pain, anorexia, sleep disorder, and constipation [5]. Heart failure not only affects the individual but also leads to an increase in infections, maximum use of health care resources, increased treatment costs, prolonged hospitalization, and a heavy burden on families and society. Finally, it reduces the quality of life of people with heart failure [6].

Heart failure increases the risk of morbidity and mortality, reduces the patient's quality of life, and imposes a significant burden on the overall healthcare system. As a result, it is necessary to evaluate the quality of life of heart failure patients [7].

Therefore, due to the increasing number of heart diseases and their effects on the lifestyle of the patient and her/his family, it seems necessary to evaluate the quality of life of heart patients. Various questionnaires have been designed in this regard, and two categories of general and specific instruments are available for this purpose. Among general measures, the Nottingham Health Profile (NHP) [8, 9], the sickness impact profile (SIP) [10], and the short form health survey (SF‐36) [11] were widely used to measure the quality of life in patients with heart failure, include general questionnaires. The disease‐specific measures for measuring quality of life in heart failure were the Minnesota Living with Heart Failure Questionnaire (MLHF‐Q) [12], Kansas City cardiomyopathy questionnaire (KCCQ) [13], left ventricular dysfunction questionnaire (LVD‐36) [14] and chronic heart failure questionnaire (CHFQ) [15]. Evidence suggests that the Minnesota Living with Heart Failure questionnaire is the most popular for heart failure patients [16]. It is worth mentioning that MLHF‐Q has been translated into 32 languages [17], and several psychometric studies of the questionnaire in different countries are available [16, 17, 18, 19, 20, 21, 22, 23, 24]. As such we also carried out a preliminary study and translated the questionnaire into the Persian and provided limited psychometric properties including a sample of 100 patients with heart failure [25]. Subsequently, the research team decided to design a new study by re‐translating the instrument and using advanced statistical analyses.

## 2 Methods

### 2.1 Minnesota Living with Heart Failure Questionnaire (MLHF‐Q)

The MLHF‐Q is a specific measure for assessing the quality of life in patients with heart failure that was developed by Rector and Cohen. The questionnaire is one of the most known questionnaire for evaluating patients' physical, mental, and social conditions. It is widely used in research and practice settings worldwide. The questionnaire contains 21 items, which are divided into three main dimensions: physical dimension (eight items), assessing the extent of the disease impact on physical abilities of patients; emotional dimension (five items) measuring patients' feelings and concerns; and social dimension (eight items), examining the impact of the disease on patients' social relations and daily activities. The questionnaire uses a 6‐point scale ranging from 0 = *no* to 5 = *very much*. The patient indicates how much heart failure has affected his or her life, with a higher score indicating a lower quality of life [12, 26].

### 2.2 Translation Procedure

After correspondence with the designer of the MLHFQ and obtaining permission (dated 15 December 2011, signed by Severine Cuchet, MAPI Research Trust), the translation process was carried out using the forward‐backward method. The English version of the questionnaire was translated into Persian by two translators fluent in both languages. The research team carefully reviewed the two Persian translations and a single version of the questionnaire was provided. This Persian version was then back‐translated into English by two translators equally proficient in both languages. A single the English version was derived from the back‐translations and then compared with the original questionnaire. Consequently, it was sent to the questionnaire's authors and the MAPI institute. After necessary corrections, the provisional version of the Persian MLHF‐Q was provided.

To assess content validity, a panel of experts reviewed the questionnaire. This panel consisted of ten experts: four cardiologists, three nurses, and three psychometricians. They were asked to suggest grammar, word choice, and item sequencing revisions. However, the experts deemed that no modifications were required. Subsequently, the questionnaire was pilot‐tested on ten patients who met the study inclusion criteria to assess face validity. The patients were asked to indicate if they needed assistance completing the questionnaire and to identify unclear or irrelevant items. The positive feedback from the patients, who reported understanding and completing the questionnaire easily, confirmed its face validity. Ultimately, the initial Persian version of the questionnaire was finalized and prepared for further psychometric evaluations [Additional file 1].

### 2.3 Psychometric Evaluation

The psychometric properties of the Persian version of the MLHF‐Q were examined using a cross‐sectional methodological approach. The inclusion criteria for the study were carefully selected to ensure the validity of our findings. Participants had to be 18 years or older, have a left ventricular ejection fraction (LVEF) of 40% by echocardiography, have at least 1 month passed since the definitive diagnosis of the disease, can read and write Persian, and are willing to participate in the study. They were also not suffering from hearing loss or any mental or cognitive disorders (The Depression, Anxiety, and Stress Scales‐21), which was used to assess the lack of mental health or cognitive disorders. Patients with acute coronary syndrome, hypothyroidism, diabetes, pulmonary hypertension, moderate to severe pulmonary disease, chronic renal failure treated with dialysis (creatinine > 2.0 mg/dl), or anemia (hemoglobin < 8.0 g/dl) were excluded.

### 2.4 Additional Measures

1. All participants were given A brief demographic questionnaire to gather data on age, marital status, education, occupation, smoking status, and functional class. The function class was evaluated by principal investigators (SE and SS) according to the New York Heart Association classification [27].
2. Depression, Anxiety, and Stress Scales‐21 (DASS‐21): It is a suitable instrument for measuring depression, anxiety, and stress and contains 21 items. Scores for the DASS‐21 subscales range from 0 to 21 and could be categorized as ‘normal,’ ‘mild,’ ‘moderate,’ ‘severe,’ and ‘very severe.’ We meticulously followed the recommended cut‐off values, an essential aspect of our methodology, to conduct an initial mental health assessment among patients [28]. The assessment was performed by two investigators (SE and SS). Patients with severe and very severe symptoms were excluded from the study. The validity and reliability of the Persian version of the questionnaire have been well‐established in prior research [29].

### 2.5 Statistical Analysis

The psychometric characteristics of the Iranian version of the MLHF‐Q were examined using the SPSS 25, AMOS 27, and JASP 0.18.3.0. Descriptive statistics, such as mean, standard deviation (SD), frequency, and percentage, were computed to summarize the data.

#### 2.5.1 Construct Validity

##### 2.5.1.1 Structural Validity

Both exploratory and confirmatory factor analyses (EFA and CFA) were performed. Since in performing both analyses, the samples should differ, as recommended, we randomly divided the dataset into two parts [30, 31, 32].

To identify the underlying factor structure, an exploratory factor analysis (EFA) with Promax rotation was conducted on the initial dataset (*n* = 326). Before the EFA, the Kaiser‐Meyer‐Olkin (KMO) measure of sampling adequacy and Bartlett's test of sphericity were employed to assess the suitability of the data for factor analysis. Factors were extracted based on eigenvalues greater than 1, communalities exceeding 0.30, and scree plot analysis [33, 34].

A confirmatory factor analysis (CFA) was conducted on a separate dataset (*n* = 201) using maximum likelihood estimation to validate the factor structure identified in the EFA. The model fit was assessed using various fit indices, including the chi‐square test (*χ*²), chi‐square/degrees of freedom ratio (*χ*²/df), comparative fit index (CFI), incremental fit index (IFI), normed fit index (NFI), Tucker‐Lewis index (TLI), relative fit index (RFI), and root mean square error of approximation (RMSEA). Model fit was considered adequate if *χ*²/df was less than 4, CFI, IFI, NFI, TLI, and RFI were more significant than 0.90, while RMSEA was less than 0.06–0.08 [33, 35].

##### 2.5.1.2 Divergent and Convergent Validity

Convergent validity assesses whether different measures of the same construct are positively related. This is evaluated by examining factor loadings, construct reliability (CR), and average variance extracted (AVE). Factor loadings, which should be above 0.50, indicate a strong relationship between items and the underlying construct. CR, a measure of internal consistency, should exceed 0.70 for satisfactory convergent validity. AVE, representing the proportion of variance explained by the construct, should be greater than 0.50 to ensure the construct is distinct and adequately measured [36].

Divergent validity ensures that a measure is distinct from related but distinct constructs. This is achieved when the maximum shared variance (MSV) is lower than the AVE and the composite reliability (CR) is above 0.70 [36].

##### 2.5.1.3 Discriminant Validity

For discriminant validity, we tested the hypothesis that patients with higher function classes, as assessed by the New York Heart Association (NYHA), would score higher on the MLHF‐Q than their counterparts, indicating lower quality of life. Thus, patients' scores on the MLHF‐Q with various function classes were compared using a one‐way analysis of variance (ANOVA).

#### 2.5.2 Reliability

1. To evaluate the internal consistency of the MLHF‐Q, Cronbach's alpha and McDonald's omega coefficients were calculated for the entire questionnaire and each factor. A coefficient of 0.70 or higher was considered acceptable [37, 38].
2. To assess test‐retest reliability, a subsample of 30 participants completed the questionnaire twice, separated by a 2‐week interval. The intraclass correlation coefficient (ICC) was calculated to measure the consistency of responses over time. An ICC value of 0.80 or higher was considered satisfactory [33, 39].

## 3 Ethics Statement

This study adhered to the ethical principles outlined in the Declaration of Helsinki and was approved by Shahed University's ethics committee(IR. Shahed. REC.1396.120). All participants or their legal guardians gave informed consent after being fully informed about the study's objectives.

## 4 Results

### 4.1 Patients' Characteristics

Five hundred twenty‐seven patients participated in this study, with their demographic characteristics summarized in Table 1. Most participants were male (62.4%) and married (77.1%). The educational level of most participants was less than that of high school (73%). The mean age of the sample was 57.90 years, with a standard deviation of 13.54 years, ranging from 25 to 85 years. Over 90% of the participants had been hospitalized for over 3 months in the preceding year.

**Table 1:** The characteristics of study participants (*n* = 527).

		Number (%)
**Gender**		
	Male	335 (62.4)
	Female	193 (37.6)
**Age group (years)**		
	25–44	60 (11.9)
	45–64	292 (54.2)
	65–85	173 (33.9)
Mean (SD)	57.90 (13.54)	—
**Educational**		
	Primary	394 (73)
	Secondary	103 (20.5)
	Higher	28 (6.5)
**Marital status**		
	Married	412 (77.1)
	Single	16 (3.5)
	Widowed	82 (16.2)
	Divorced	16 (3.4)
**Employment status**		
	Housewife	150 (27.1)
	Employed	63 (12.4)
	Retired	97 (18.6)
	Unemployed	216 (41.9)
**Etiology of chronic heart failure**		
	Ischemic	165 (32)
	Chagas disease	108 (20)
	Idiopathic	77 (15)
	Multifactorial	75 (14)
	Hypertensive	37 (7)
	Valvular	32 (6)
	Other (congenital, viral, peripartum, and others)	32 (6)
**Function class** a		
	I	180 (32.4)
	II	155 (30)
	III	111 (21.9)
	IV	81 (15.7)
**Charlson comorbidity index**		
	1	173 (32.8)
	2	162 (30.7)
	3	100 (19.1)
	> 3	92 (17.4)
**Hospitalization in previous year**		
	Less than three months	48 (9.5)
	More than three months	479 (90.5)
**Smoking**		
	Yes	106 (21.4)
	No	330 (61)
	Quit smoking	91 (17.6)

^a^ New York Heart Association (NYHA) Classes: Class I: Patient is comfortable with ordinary physical activity, but elevated activity causes symptoms, such as fatigue and shortness of breath. Class II: Patient is comfortable at rest, but ordinary physical activity causes symptoms. Class III: Even light activity causes patient fatigue, heart palpitation, or shortness of breath. Class IV: Patient shows symptoms at rest, and any physical activity only increases the discomfort.

### 4.2 Exploratory Factor Analysis

The Kaiser‐Meyer‐Olkin (KMO) measure of sampling adequacy was found to be 0.963, indicating that the data was suitable for factor analysis. Bartlett's test of sphericity was statistically significant (*χ*² = 7782.341, *p* < 0.001), further confirming the factorability of the data. A factor analysis was conducted to extract factors. Three factors with eigenvalues greater than 1 were identified, collectively explaining 79.46% of the total variance. Of the 21 items, 18 had factor loadings exceeding 0.70, indicating strong relationships with the underlying factors. The remaining four items had loadings between 0.49 and 0.70, suggesting moderate relationships (Table 2).

**Table 2:** The results obtained from the exploratory factor analysis for the Minnesota living with heart failure questionnaire‐MLHF‐Q (*n* = 326).

Items	F1	F2	F3
5. Making you're going places away from home difficult?	**0.930**	0.020	0.088
4. Making your working around the house or yard difficult?	**0.924**	0.011	0.137
6. Making your sleeping well at night difficult?	**0.916**	0.035	0.108
		0.106	0.020
7. Making your relating to or doing things with your friends or family difficult?	**0.838**	0.016	0.138
12. Making you short of breath?	**0.806**	0.080	0.138
3. Making your walking about or climbing stairs difficult?	**0.776**	0.177	0.128
2. Making you sit or lie down to rest during the day?	**0.754**	0.181	0.254
13. Making you tired, fatigued, or low on energy?	**0.714**	0.137	−0.004
21. Making you feel depressed?	0.115	**0.967**	−0.037
18. Making you feel a loss of self‐control in your life?	0.053	**0.943**	0.088
17. Making you feel you are a burden to your family or friends?	0.041	**0.835**	0.137
20. Making it difficult for you to concentrate or remember things?	0.057	**0.763**	0.108
19. Making you worry?	0.153	**0.742**	0.020
9. Making your recreational pastimes, sports, or hobbies difficult?	0.134	0.126	**0.881**
8. Making your working to earn a living difficult?	0.029	0.033	**0.772**
10. Making your sexual activities difficult?	0.048	0.001	**0.748**
14. Making you stay in a hospital?	0.033	0.232	**0.710**
11. Making you eat less of the foods you like?	−0.171	0.163	**0.709**
1. Causing swelling in your ankles or legs?	0.115	0.173	**0.645**
16. Giving you side effects from treatments?	0.198	0.425	**0.511**
15. Costing you money for medical care?	0.134	0.0365	**0.493**
*Eigenevalue*	11.27	3.35	2.06
*% Variance*	53.67	15.96	9.83

*Note:* F1, Physical; F2, Emotional; F3, Socio‐economic impairments.

### 4.3 Confirmatory Factor Analysis

**Figure 1:**
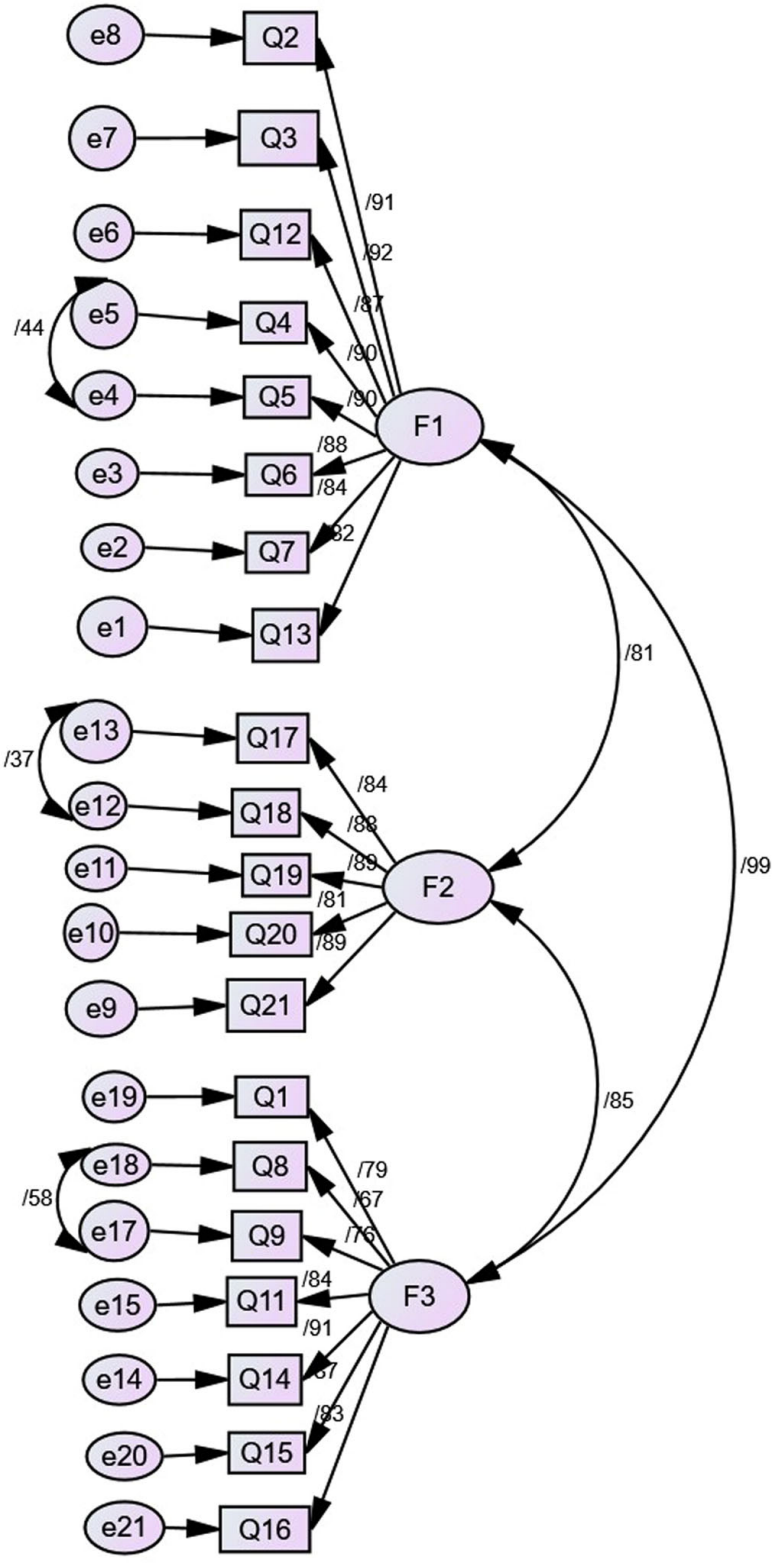
First‐order confirmatory factor analysis based on the 3‐factor model, including correlation between factors, factor loadings, and errors.

**Figure 2:**
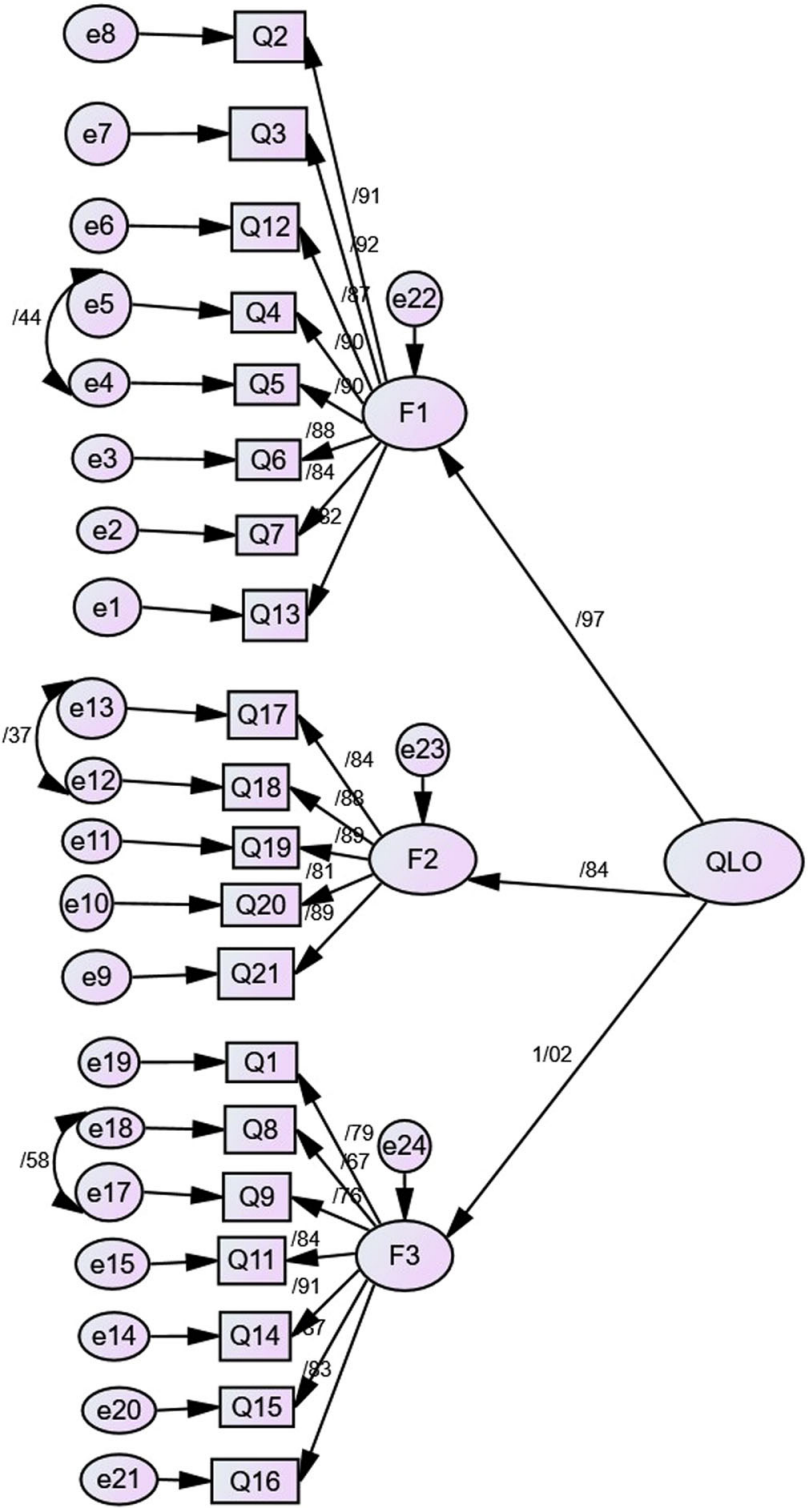
Second‐order confirmatory factor analysis based on the 3‐factor model, including correlation between factors, factor loadings, and errors.

A confirmatory factor analysis (CFA) was conducted to validate the factor structure identified in the exploratory factor analysis. Several modifications were made to improve model fit. First, item 10 was removed due to its low factor loading. Second, correlated error terms were specified between items 4 and 5 (e 4 and e5), items 17 and 18 (e12 and e13), and items 8 and 9 (e17 and e18). The modified model's fit was assessed using various fit indices. The model exhibited acceptable fit, with a *χ*² value of 200.606, a *χ*²/df ratio of 2.33, a CFI of 0.92, an NFI of 0.88, an IFI of 0.93, a TLI of 0.91, and an RMSEA of 0.08. Detailed results are presented in Table 3 and Figure 1. A second‐order factor analysis was performed to explore the hierarchical structure of the MLHFQ further. This analysis examined whether the three first‐order factors (physical, emotional, and social) were subsumed under a single, higher‐order factor (Figure 2).

**Table 3:** The range of acceptable fit indexes and the results obtained for confirmatory factor analysis‐CFA (*n* = 201).

Indexes	Cut off value	CFA
**CMIN/df**	< 3	2.33
***p* value** a	≥ 0.05	< 0.0001
**TLI**	≥ 0.95	0.91
**IFI**	≥ 0.90	0.93
**NFI**	≥ 0.90	0.88
**CFI**	≥ 0.95	0.92
**PCFI**	≥ 0.5	0.76
**PNFI**	≥ 0.5	0.72
**RMSEA**	≤ 0.06 to 0.08	0.08

Abbreviations: CFI, comparative fit index; CMIN/DF, minimum discrepanscy funcation by degrees of freedom divided; IFI, incremental fit index; PCFI, parsimonious comparative fit index; PNFI, parsimonious normed fit index; RMSEA, root mean square error of approximation; TLI, tucker –lewis index.

^a^ Derived from Chi‐square test (*χ*²), a measure for fit indexes in structural equation modeling (SEM).

### 4.4 Convergent and Divergent Validity

Convergent validity was assessed using each factor's average variance extracted (AVE). As shown in Table 4, the AVE for all constructs exceeded the recommended threshold of 0.50, and the composite reliability (CR) for each construct was above 0.70, indicating strong internal consistency. Thus, the measurement model demonstrates acceptable convergent validity, confirming convergence between the constructs. However, divergent validity was not supported, as the maximum shared variance (MSV) values exceeded the AVE values.

**Table 4:** Convergent and divergent values for the Minnesota living with heart failure questionnaire (MLHF‐Q).

	MSV	AVE	CR
Physical	0.97	0.77	0.96
Emotional	0.72	0.75	0.93
Socio‐economic impairments	0.97	0.66	0.93

Abbreviations: AVE, average variance extracted; CR, construct reliability; MSV, maximum shared variance.

### 4.5 Discriminant Validity

The discriminative validity of the MLHF‐Q was confirmed through ANOVA models analyzing variations in NYHA scores. The physical dimension and the total score effectively differentiated between the three groups, with statistically significant results (*p* < 0.001) as presented in Table 5.

**Table 5:** Comparison of the Minnesota living with heart failure questionnaire (MLHF‐Q) scores by functional class (Known‐groups comparison)^a^.

	I (*n* = 111)	II (*n* = 96)	III/IV (*n* = 119)	
	Mean (SD)	Mean (SD)	Mean (SD)	*p*
**Physical**	20.58 (13.72)	22.54 (10.37)	103.76 (23.42)	< 0.0001
**Emotional**	9.50 (7.95)	9.21 (6.70)	34.16 (18.77)	0.002
**Socio‐economic impairments**	17.32 (3.34)	27.4 (6.21)	65.1 (15.03)	< 0.0001
**Total**	44.18 (24.58)	47.05 (18.53)	204.83 (54.93)	< 0.0001

^a^ Higher scores indicate lower quality of life.

### 4.6 Reliability

The Cronbach's alpha and McDonald's Omega were estimated at 0.97 for the whole MLHF‐Q, The Cronbach Coefficient alpha for the subscales was (0.96, 0.93, and 0.93), and McDonald's Omega (0.96, 0.93, and 0.94) respectively. The overall ICC for the MLHF‐Q was 0.98, indicating excellent stability for the test. The findings are shown in Table 6.

**Table 6:** The Cronbach's alpha and the Intraclass Correlation Coefficients (ICC) for the Persian version of the Minnesota living with heart failure questionnaire (MLHF‐Q).

	Number of items	Cronbach's alpha (*n* = 326)	MC Doland's omega	ICC; 95% CI (*n* = 30)	*p*
**Physical**	8	0.96	0.96	0.98 (0.99–0.99)	< 0.0001
**Emotional**	5	0.93	0.93	0.97 (0.91–0.92)	< 0.0001
**Socio‐economic impairments**	8	0.93	0.94	0.93 (0.82‐0.96)	< 0.0001
**Total score**	21	0.97	0.97	0.98 (0.99–0.99)	< 0.0001

Abbreviation: ICC, Intraclass correlation coefficient.

## 5 Discussion

This study sought to evaluate the psychometric properties of the Persian version of the Minnesota Living with Heart Failure Questionnaire (MLHF‐Q) within an Iranian population. The findings revealed that the Persian version of the MLHF‐Q has robust psychometric properties, including a well‐supported three‐factor structure encompassing physical, emotional, and social dimensions. The confirmatory factor analysis (CFA) demonstrated satisfactory model fit indices, confirming the questionnaire's structural validity. Reliability measures, including Cronbach's alpha and intraclass correlation coefficient, indicated high internal consistency and stability.

Although previously we did a small scale study to translate and validate the MLHF‐Q in Iran [25], The current study utilized a larger sample size (527 patients), allowing for more precise statistical results and greater confidence in findings. The larger sample size, and using both like exploratory factor analysis (EFA) and CFA, provided a better chance of uncovering underlying structures within the questionnaire. Such analyses were essential for confirming the existence of three factors (physical, emotional, and social) in the structure of the MLHF‐Q lending support to it use in countries such as Iran. However, by leveraging a larger sample size and advanced analyses, the current study has demonstrated the heightened significance of the social dimension in Iran, likely attributable to the culture's strong emphasis on family and social support for patients. This finding underscores the potential for cultural variations in the performance of the MLHFQ across different countries and opens avenues for broader cross‐cultural comparisons. Additionally, we performed test‐retest reliability, demonstrating the stability of responses over time and supporting the questionnaire's high reliability. Based on the more precise results obtained, the study recommends the Persian version of the MLHFQ as a reliable questionnaire for assessing the quality of life in heart failure patients in Iran and other Persian‐speaking communities.

The results of this study align with previous research on the MLHF‐Q conducted in various cultural contexts, which generally supports a two or three‐factor structure. For instance, while Rector and Cohn initially identified two primary physical and emotional dimensions within the MLHF‐Q [12, 40], other studies have found evidence for an additional social factor [16, 17, 20, 24, 41, 42, 43, 44].

Confirming a three‐factor structure within the Iranian population extends the generalizability of the MLHF‐Q across diverse cultural settings. It suggests that the experiences of heart failure patients, as captured by the questionnaire, have universal aspects, particularly regarding how physical limitations, emotional distress, and social impairments interplay to affect the quality of life [17]. These findings reinforce the utility of the MLHF‐Q as a comprehensive questionnaire for assessing the quality of life in heart failure patients globally, including within non‐Western contexts such as Iran.

The results obtained for the CFA were satisfactory. The fit indices and factor loadings indicate that the three‐factor version has adequate structural validity and fit to the model proposed by several studies [12, 17, 24, 33, 45, 46, 47].

The MLHF‐Q total score and its dimensions effectively differentiate between various levels of heart failure severity, as assessed by the NYHA functional class. Previous research on adult populations has consistently shown that the physical dimension exhibits superior discriminatory ability compared to other dimensions. For example, the Dutch version of the MLHF‐Q demonstrated that while the total score and all dimensions could distinguish between NYHA Classes I, II, and III, the physical dimension had the most significant effect size [48]. Similarly, Bennett et al. found that the physical dimension could differentiate among all four functional classes. In contrast, the total score and emotional dimension failed to distinguish between Classes III and IV [49]. Given that NYHA functional classes are categorized based on symptom severity, it is expected that the physical dimension of the MLHF‐Q would have a stronger discriminatory capacity. However, the ability of the MLHF‐Q to consistently differentiate across all functional classes remains to be determined [50].

The Cronbach's alpha was calculated 0.97 for the Persian version of the MLHF‐Q which was similar to reported results of German (0.94), Greek (0.97), Portuguese (0.97), Korean (0.95), Spanish (0.94), Australia (0.91), Czech (0.90), France (0.91), Hungary (0.91), Israel (0.93), and Chinese (0.95) versions [16, 17, 18, 20, 22, 41, 51, 52, 53]. Also, reviewing this questionnaire in a sample collected from 21 countries, the reliability of the questionnaire was reported to be 0.90 [17]. The stability after 2 weeks is also reported to be appropriate. The stability after 2 weeks is also reported to be appropriate. The ICC value was 0.98, consistent with findings from other observations, such as studies that reported an ICC of 0.80 [16], 0.88 [21], 0.83 [53], 0.89 [42], 0.73 [48], and 0.80 [22].

The robust psychometric properties observed in this study could be attributed to the rigorous translation and cultural adaptation processes undertaken. The forward‐backward translation methodology, expert panel reviews, and pre‐testing ensured that the Persian version of the MLHF‐Q was linguistically and culturally appropriate for the target population. This careful adaptation likely contributed to the high reliability and validity indices observed [54].

Unexpectedly, the study found that the social dimension was significantly relevant in the Iranian context, perhaps more so than reported in some Western studies. This may be explained by cultural differences in social structures and the role of family and community in patient care in Iran. In Iranian culture, social support and family involvement are integral to managing chronic illnesses such as heart failure, which might explain the prominence of the social dimension in this study.

Finally, 90% of the sample in this study were hospitalized for more than 3 months in the previous year. Prolonged hospitalizations could influence the quality of life in patients with heart failure and may increase mortality. However, more extended hospitalizations occur due to clinical, social, cultural, and economic factors [55, 56, 57]. Different samples might lead to different psychometric properties for the instrument. Such a hypothesis should be examined in future studies, and to prevent misleading results, it might be better to add extra items to the questionnaire if the sample consists of patients with a history of lengthy hospitalization.

## 6 Practical, Theoretical, and Clinical Significance

The findings from this study have important practical implications. Clinicians and researchers in Iran and other Persian‐speaking regions can now confidently use the Persian version of the MLHF‐Q to assess the quality of life in heart failure patients. This questionnaire can facilitate better patient monitoring, allowing for more personalized care strategies that address the multifaceted impact of heart failure on patients' lives. Theoretically, this study contributes to the broader literature on quality‐of‐life assessments in chronic illness by validating the MLHF‐Q within a new cultural context. It supports the notion that while cultural nuances exist, certain aspects of the heart failure experience are universal, validating the MLHF‐Q's cross‐cultural applicability.

## 7 Future Research Directions

Future research could explore several avenues. First, longitudinal studies could assess how the MLHF‐Q scores change with disease progression or in response to interventions. Additionally, further research could examine the questionnaire predictive validity concerning clinical outcomes such as hospitalization rates and mortality. Expanding the validation to other Persian‐speaking populations in different regions would also enhance the generalizability of the findings. Lastly, qualitative studies explore in‐depth how cultural factors influence the perception of heart failure‐related quality of life, providing insights that could refine the MLHF‐Q or inspire the development of supplementary instruments.

## 8 Limitations

This study has a few limitations that should be acknowledged. The large sample was drawn from a single setting, which may limit the generalizability of the findings to all Iranian heart failure patients. The exclusion of patients with severe co‐morbid conditions also limits the applicability of the findings to more diverse patient groups. Future studies could address these limitations by incorporating more diverse samples and ensuring that the questionnaire is responsive to changes after clinical interventions.

## 9 Conclusions

The Persian version of the MLHF‐Q has demonstrated strong psychometric properties, making it a reliable and valid questionnaire for assessing the quality of life in heart failure patients within the Iranian context. Despite some limitations, this study provides a solid foundation for the continued use and further exploration of the MLHF‐Q in Persian‐speaking populations.

## Author Contributions

**Majideh Heravi‐Karimooi:** conceptualization; investigation; methodology; validation; supervision; writing–review & editing; writing–original draft. **Razieh Bandari:** conceptualization; investigation; methodology; software; project administration; resources; supervision; data curation; visualization; validation; funding acquisition; writing–original draft; writing–review & editing; formal analysis. **Somayeh Eskandari:** data curation; writing–review & editing; writing–original draft. **Sahar Semnani:** data curation; writing–review & editing; writing–original draft. **Nahid Rejeh:** conceptualization; writing–original draft; writing–review & editing; methodology. **Ali Montazeri:** conceptualization; project administration; resources; supervision; data curation; writing–review & editing; writing–original draft; investigation.

## Conflicts of Interest

The authors declare no conflict of interest.

## Transparency Statement

The lead author Razieh Bandari, Ali Montazeri affirms that this manuscript is an honest, accurate, and transparent account of the study being reported; that no important aspects of the study have been omitted; and that any discrepancies from the study as planned (and, if relevant, registered) have been explained.

## Supporting information

Additional file 1: The Persian version of the Minnesota living with heart failure questionnaire (MLHF‐Q) (docx).

## Data Availability

The data that support the findings of this study are available from the corresponding author upon reasonable request. The authors confirm that the data supporting this study's findings are available in the article and its supplementary materials.
