# Supplementary material for: Validation Study of the Iranian Version of Minnesota Living With Heart Failure Questionnaire (MLHF‐Q): A Cross‐Sectional Study
Source: Health Sci Rep. 2025 Feb 3;8(2):e70396. doi: 10.1002/hsr2.70396 (PMC11790469; doi:10.1002/hsr2.70396)
Supplement: Supplementary file 1 — Additional file 1: The Persian version of the Minnesota living with heart failure questionnaire (MLHF‐Q) (docx). [file HSR2-8-e70396-s001.doc]

**بسمه تعالي**

**پرسشنامه کیفیت زندگی بیماران نارسایی قلبی مینسوتا**

**در سوالات زیر، از شما سوال می شود نارسایی قلبی(وضعیت قلبی) شما چقدر بر زندگیتان در یک ماه گذشته تاثیر گذاشته است. بعد از مطالعه هر سوال، دور یکی از اعداد 0،1،2،3،4،5 که نشان دهنده میزان تاثیرپذیری زندگی شما از نارسایی قلبی شما است دایره بکشید. اگر سوالی درباره شما صدق نمی کند، دور عدد صفر دایره بکشید.**

آیا در طی ماه گذشته نارسایی قلبی، توانسته است از طریق موارد زیر سبب شود نتوانید آن گونه که می خواهید زندگی کنید؟

|  | | **خير** | **خيلي كم** |  |  |  | **خيلي زياد** |
| --- | --- | --- | --- | --- | --- | --- | --- |
| 1 | ايجاد تورم در قوزک یا ساق پاهايتان | 0 | 1 | 2 | 3 | 4 | 5 |
| 2 | مجبور کردن شما به نشستن یا دراز کشیدن برای استراحت در طول روز | 0 | 1 | 2 | 3 | 4 | 5 |
| 3 | ایجاد مشکل در قدم زدن يا بالا رفتن از پله ها | 0 | 1 | 2 | 3 | 4 | 5 |
| 4 | ایجاد مشکل در انجام کارهای منزل يا حياط شما | 0 | 1 | 2 | 3 | 4 | 5 |
| 5 | ایجاد مشکل در رفتن به مسافت های دور از منزل شما | 0 | 1 | 2 | 3 | 4 | 5 |
| 6 | ایجاد مشکل در خواب مطلوب شبانه | 0 | 1 | 2 | 3 | 4 | 5 |
| 7 | ایجاد مشکل در ارتباط داشتن یا انجام دادن كار ها با دوستان و يا اعضای خانواده | 0 | 1 | 2 | 3 | 4 | 5 |
| 8 | ایجاد مشکل دركار كردن و کسب درآمد | 0 | 1 | 2 | 3 | 4 | 5 |
| 9 | ایجاد مشکل در تفريح کردن، ورزش و یا انجام امور مورد علاقه | 0 | 1 | 2 | 3 | 4 | 5 |
| 10 | ایجاد مشکل درانجام فعاليت جنسي | 0 | 1 | 2 | 3 | 4 | 5 |
| 11 | مجبور کردن شما به کمتر خوردن غذاهايي كه دوست داريد | 0 | 1 | 2 | 3 | 4 | 5 |
| 12 | ایجاد تنگي نفس در شما | 0 | 1 | 2 | 3 | 4 | 5 |
| 13 | ایجاد احساس خستگی، ضعف یا کاهش انرژی شما | 0 | 1 | 2 | 3 | 4 | 5 |
| 14 | مجبور کردن شما به بستری شدن در بيمارستان | 0 | 1 | 2 | 3 | 4 | 5 |
| 15 | ایجاد هزينه مراقبت های پزشكي شما | 0 | 1 | 2 | 3 | 4 | 5 |
| 16 | ایجاد عوارض جانبي ناشی از درمان در شما | 0 | 1 | 2 | 3 | 4 | 5 |
| 17 | ایجاد احساس سربار بودن برای خانواده يا دوستان در شما | 0 | 1 | 2 | 3 | 4 | 5 |
| 18 | ایجاد احساس فقدان خود کنترلی در زندگي | 0 | 1 | 2 | 3 | 4 | 5 |
| 19 | ایجاد نگرانی در شما | 0 | 1 | 2 | 3 | 4 | 5 |
| 20 | ایحاد مشکل در تمركز و يا به يادآورن چیز ها | 0 | 1 | 2 | 3 | 4 | 5 |
| 21 | ایجاد احساس افسردگی در شما | 0 | 1 | 2 | 3 | 4 | 5 |
